# LATENT PROFILE ANALYSIS OF SOCIAL PARTICIPATION IN STROKE SURVIVORS WITH LIMB DYSFUNCTION: A MIXED-METHODS STUDY

**DOI:** 10.2340/jrm.v58.44832

**Published:** 2026-01-07

**Authors:** Xuan ZHOU, Ying WANG, Lanshu ZHOU

**Affiliations:** 1 School of Nursing, Naval Medical University, Shanghai; 2 Department of Nursing, Shanghai First Rehabilitation Hospital, Shanghai, China

**Keywords:** stroke, social participation, latent profile analysis, mixed-methods study, limb dysfunction

## Abstract

**Objective:**

This study aimed to identify profiles of social participation among stroke survivors with limb dysfunction and explore the factors influencing these profiles.

**Design:**

A convergent mixed-methods design.

**Methods:**

The quantitative phase involved 499 participants recruited from 5 neurorehabilitation centres in Shanghai between December 2023 and June 2025. Participants completed measures including the Utrecht Scale for Evaluation of Rehabilitation-Participation (USER-P), the Connor-Davidson Resilience Scale (CD-RISC-10), and the Modified Rankin Scale (mRS). Qualitative semi-structured interviews were performed with 16 participants to explore lived experiences of social participation.

**Results:**

Latent profile analysis revealed 4 distinct social participation profiles: “Active Integration”, “Contented Conservatism”, “Cautious Conservatism”, and “Alienated Disengagement”. A multivariate analysis identified age, resilience, and physical function as significant predictors of profile membership. Qualitative findings uncovered 2 core emotional experiences (a sense of loss vs a sense of rebuilding) and 3 behavioural patterns (activist, conservative, alienated), which effectively explained the quantitative profiles’ characteristics and their underlying mechanisms. The integration of data provided a nuanced person-centred framework depicting the heterogeneity in post-stroke social participation.

**Conclusion:**

Social participation among stroke survivors is heterogeneous and can be classified into 4 distinct profiles shaped by the interplay of physical function, resilience, and sociodemographic factors. The findings underscore the necessity of implementing profile-specific, stepped-care interventions for effectively enhancing post-stroke social participation.

## LAY ABSTRACT

Stroke often causes extremity disability, significantly impacting survivors’ ability to engage in social activities. This study aimed to understand the different ways stroke survivors participated in society. We surveyed 499 stroke survivors and interviewed 16 others. We identified 4 distinct patterns: “Active Integration” (actively engaged), “Contented Conservatism” (less active but satisfied), “Cautious Conservatism” (less active and dissatisfied), and “Alienated Disengagement” (largely withdrawn). The pattern a stroke survivor fell into was influenced not just by physical function but also by age, psychological resilience, and emotional outlook – particularly whether they focused on loss or on rebuilding their life after stroke. These findings highlight that personalized support plans, tailored to an individual’s specific needs and mindset, are crucial for effectively helping stroke survivors regain social connection and improve their quality of life.

Stroke is a leading cause of long-term disability worldwide, affecting millions of people each year. According to the World Health Organization, there are over 12 million new cases of stroke annually (1). In China, the disease burden is substantial, with an estimated 17.8 million stroke survivors, 3.4 million new cases, and 2.3 million deaths in 2020 (2). Furthermore, approximately 12.5% of stroke survivors were left with significant disability. These conditions, particularly limb dysfunction, profoundly impact patients’ social participation and quality of life (3). Social participation, which encompasses roles, activities, and interpersonal connections, is a critical determinant of post-stroke recovery and psychosocial well-being (4, 5). However, stroke survivors frequently face multifaceted challenges in social reintegration (6), which can further hinder physical recovery, exacerbate psychological distress, and increase mortality risk (7, 8).

Existing evidence suggests that social participation in stroke survivors is influenced by a complex interplay of factors, including mobility limitations, resilience, and environmental barriers (9–11). Despite this understanding, interventions targeting these factors, such as exercise-based programmes, self-management strategies, and occupational therapy, have yielded inconsistent outcomes in improving social participation (12, 13). This discrepancy may stem from the heterogeneous nature of post-stroke social participation patterns, which are often overlooked in standardized, one-size-fits-all intervention approaches. A paradigm shift towards individualized strategies that are tailored to distinct participation profiles is urgently needed (14).

To address this gap, our study employs a convergent mixed-methods design to provide a comprehensive understanding of this heterogeneity. This approach integrates: (*i*) latent profile analysis (LPA), a person-centred statistical approach that identifies homogeneous subgroups within heterogeneous populations based on social participation metrics (frequency, restrictions, and satisfaction); and (*ii*) qualitative thematic analysis, which captures the lived experiences and emotional responses that quantitative tools cannot fully elucidate (15, 16). Through this dual-focus lens, this study aims to identify latent profiles of social participation among stroke patients with limb dysfunction and examine potential factors associated with distinct participation profiles.

## METHODS

This study adopted a convergent mixed-methods design to comprehensively explore the profiles of social participation. This approach allowed for the triangulation of quantitative patterns with qualitative experiences, providing a more complete understanding, though it also presented challenges in data integration. The study was conducted in accordance with the Declaration of Helsinki, with the protocol receiving ethical approval from the Ethics Committee of Shanghai First Rehabilitation Hospital (2022-01-001). Written informed consent was obtained from all study participants. This study adheres to the Reporting Guidelines for Mixed Methods Research in Rehabilitation Health Sciences (17).

### Participants

Participants were recruited from 5 neurorehabilitation departments in Shanghai, China, between December 2023 and June 2025. The inclusion criteria were defined as follows: (*i*) diagnosis of stroke confirmed by CT or MRI; (*ii*) age ≥18 years old; (*iii*) stable vital signs and absence of other serious comorbidities, such as cancer or organ failure; (*iv*) disease duration of at least 3 months; (*v*) presence of limb dysfunction with a modified Rankin Scale (mRS) score between 1 and 4; and (*vi*) voluntary participation in this study. Exclusion criteria included: (*i*) language communication disorders; (*ii*) cognitive impairment, defined as a Montreal Cognitive Assessment (MoCA) score < 26; and (*iii*) severe emotional disorders. Based on methodological recommendations that latent profile analysis (LPA) requires a sample size between 300 and 500 (18), a total of 499 stroke survivors successfully completed the survey, yielding an effective response rate of 92.1%. For the qualitative phase, participants were selected based on the same eligibility criteria using a purposive sampling strategy. Between March 2025 and June 2025, stroke patients were recruited from both neurorehabilitation departments and community settings. The sampling aimed to maximize variation across key characteristics such as age, educational background, and disease course. Data saturation was determined to be achieved after the 16th interview, at which point no new themes emerged.

### Measurements

Data on sociodemographic and clinical characteristics were collected using a purpose-designed questionnaire, including age, gender, educational level, stroke type, and time after onset.

Social participation was assessed using the Utrecht Scale for Evaluation of Rehabilitation-Participation (USER-P), which comprises 32 items across 3 subscales: Participation Frequency, Participation Restriction, and Participation Satisfaction (19). The Frequency subscale contains 2 parts: 4 items rated from 0 (never) to 5 (≥36 h/week) and 7 items rated 0 (never) to 5 (≥19 times/month). The Restriction subscale includes 11 items scored from 0 (impossible) to 3 (no difficulty), and the Satisfaction subscale consists of 10 items rated 0 (very dissatisfied) to 4 (very satisfied). The USER-P does not yield a total score; instead, raw scores for each subscale are converted to a normalized score ranging from 0 to 100 using specific algorithms. Higher scores indicate higher social participation frequency, fewer participation restrictions, and greater satisfaction with participation (20). The Chinese version shows good reliability (Cronbach’s α: 0.704–0.861; test–retest: 0.734–0.832) and validity (21).

The 10-item Connor–Davidson Resilience Scale (CD-RISC-10) was utilized to evaluate the resilience level of stroke patients. Originally developed by Connor and Davidson in 2003 (22), the scale comprised 25 items distributed across 5 dimensions. Subsequently, Campbell-Sills and Stein refined it into a condensed 10-item version in 2007, enhancing its conciseness and user-friendliness. The CD-RISC-10 employs a 4-point Likert response format, ranging from 1 (not true at all) to 4 (true nearly all the time), yielding a total score that spans from 10 to 40. Higher total scores are indicative of a greater degree of resilience (23). The CD-RISC-10 exhibits strong psychometric properties, including a Cronbach’s α coefficient of 0.94, a split-half reliability coefficient of 0.89, and item-total correlations varying between 0.74 and 0.81 (23, 24).

Functional status was assessed using the mRS. The mRS was initially developed by Rankin in 1957 and was later modified and optimized by Warlow in 1988 to improve its comprehensiveness; this revised version is now the standard tool employed in clinical practice (25). The mRS utilizes a 7-level scoring system to evaluate functional independence in stroke patients, with specific criteria for each grade: a score of 0 denotes no symptoms; 1 indicates no significant disability despite symptoms; 2 corresponds to slight disability; 3 represents moderate disability; 4 indicates moderately severe disability; 5 reflects severe disability; and 6 signifies death.

### Data collection

*Quantitative data collection.* Printed advertisements outlining the research project were distributed across 5 neurorehabilitation facilities and 1 community setting to invite eligible stroke patients to participate. All prospective volunteers were provided with comprehensive explanations regarding the study’s purpose, potential benefits, and possible risks. Each candidate was explicitly advised of their rights and responsibilities, including the unconditional right to withdraw at any time without impact on their medical care. Formal written consent was obtained from every participant prior to inclusion. Questionnaires were administered face-to-face using both paper-based forms and digital platforms such as Questionnaire Star. Before distribution, researchers clearly explained the instructions and key points for completion. After participants finished, each questionnaire was reviewed for missing responses. Where omissions were identified, the reasons were explored, and researchers provided assistance to complete any unfinished items when appropriate. For those with visual impairments or limited literacy, items were read aloud and responses were recorded on their behalf based on their verbal answers. A supportive rapport was maintained with all participants throughout the process, and contact information was gathered to enable future follow-up. A total of 545 individuals participated in the survey. Among them, 18 were excluded due to patterned responding, 26 were omitted for extensive missing data, and 2 voluntarily withdrew during the process, resulting in 499 valid responses.

*Qualitative data collection.* The qualitative phase employed one-on-one, in-depth, semi-structured interviews. The participants for this phase were a distinct group, separately recruited and not involved in the prior quantitative survey. This approach facilitated face-to-face dialogue between the researcher and participant, aimed at garnering rich insights into individuals’ experiences, emotions, and perspectives regarding social participation after stroke. An interview guide was developed in advance to ensure coverage of key topics while allowing flexibility to adapt to the flow of conversation. Participants were also encouraged to introduce relevant issues or questions they deemed important. Key interview topics included: current well-being; personal reflections on the stroke event; modes and extent of social participation; emotional and experiential dimensions of participation; and perceived strategies to enhance social engagement. Each interview lasted approximately 40 to 60 min to balance depth of content with participant comfort.

### Data analysis

*Quantitative data analysis.* Statistical analyses were conducted using Mplus 8.0 (https://www.statmodel.com/) and SPSS 27.0 (IBM Corp, Armonk, NY, USA). Categorical data are presented as frequencies and percentages, while continuous data are summarized as mean and standard deviation for normally distributed variables or median and interquartile range for non-normally distributed variables. LPA was performed in Mplus 7.4 to identify subtypes of social participation among stroke patients with limb dysfunction, using the mean scores of the 3 USER-P subscales as manifest variables. The analysis commenced with a one-profile model and progressively increased the number of profiles. Model selection was guided by fit indices, including information criteria (AIC, BIC, and aBIC; lower values indicate better fit), likelihood ratio tests (LMRT and BLRT; *p* < 0.05 suggests significant improvement over the previous model), and entropy (range 0–1; higher values indicate clearer classification). The resulting profiles were interpreted and named based on their distinctive characteristics. Pairwise comparisons of the 3 subscales across profiles were conducted using the Kruskal–Wallis H test. The profiles were then treated as a dependent variable in univariate analyses involving χ^2^ and *t*-tests to examine associations with potential predictors. Variables that reached statistical significance (*p* < 0.05) in univariate analyses were entered as independent variables into a multinomial logistic regression model. A two-tailed *p*-value < 0.05 was considered statistically significant throughout.

*Qualitative data analysis.* To avoid potential bias from quantitative results, qualitative themes were extracted first. Interviews were transcribed verbatim within 24 h and verified by 2 researchers to ensure accuracy. Transcriptions adhered to principles of completeness, timeliness, and multiple backups. Nonverbal behaviours such as pauses, emotional expressions, and body language were documented in memos. Participant demographics were managed in Excel (Microsoft Corp, Redmond, WA, USA). Thematic analysis followed Colaizzi’s phenomenological approach, a widely established method for deriving meaningful themes from qualitative data to illuminate participants’ experiences, emotions, and behaviours (26). The process included: (*i*) immersion in the data, (*ii*) identification of significant statements, (*iii*) coding and grouping of meanings, (*iv*) development and refinement of themes, and (*v*) validation of findings through reflection and comparison with existing literature to ensure interpretive rigour.

## RESULTS

### General characteristics of study participants

A total of 499 participants completed the survey. The mean age was 68.25 years (SD = 10.44). Among the participants, 298 (59.7%) were male and 372 (74.5%) had ischaemic stroke. The mean time since stroke onset was 12.13 months (SD = 16.33). The modified Rankin Scale (mRS) scores were distributed as follows: 7.0% scored 1, 32.3% scored 2, 42.7% scored 3, and 18.0% scored 4. Detailed characteristics are presented in Table I.

**Table I:** Sociodemographic and clinical characteristics of participants (*n* = 499)

Variables	
Age, *n* (%)	68.25 (10.44)
Gender, *n* (%)	
Male	298 (59.7)
Female	201 (40.3)
Educational background, *n* (%)	
Primary school and below	161 (32.3)
Middle school	276 (55.3)
University and above	62 (12.4)
Spousal status, *n* (%)	
Yes	430 (86.2)
No	69 (13.8)
Employed before disease onset, *n* (%)	
Yes	95 (19.0)
No	404 (81.0)
Caregiver, *n* (%)	
No caregiver	29 (5.8)
Care provided by relatives	236 (47.3)
Care provided by nursing assistant	234 (46.9)
Family financial situation, *n* (%)	
Poor	31 (6.2)
Average	302 (60.5)
Good	166 (33.3)
Stroke type	
Ischaemic, *n* (%)	372 (74.5)
Haemorrhagic, *n* (%)	127 (25.5)
Disease course, months, mean ± SD	12.13 ± 16.33
mRS, points, *n* (%)	
1	35 (7.0)
2	161 (32.3)
3	213 (42.7)
4	90 (18.0)

### Potential profile analysis of social participation in stroke survivors

Starting with a one-profile model, we incrementally increased the number of profiles using the 3 USER-P subscale scores as indicators. The fit indices for competing models are presented in Table II. When the potential profiles of social participation were 5, the LMR value had no statistical significance (*p* = 0.497). When the potential profiles of social participation were 4, the entropy value was 0.866, and both the LMR value and the BLRT value were statistically significant (*p≤*0.05). The AIC, BIC, and aBIC values were all smaller than those when the potential profiles were 2 or 3. Finally, the model with 4 latent profiles was determined to be the best fitting.

**Table II:** Fit indices for latent profile analysis of social participation among stroke survivors with limb impairment

Profile	k	AIC	BIC	aBIC	Entropy	*p*-value		Profile proportions
						LMR	BLRT	
1	6	13,377.285	13,402.561	13,383.516	–	–	–	1.000
2	10	13,093.210	13,135.336	13,103.595	0.741	0.0005	< 0.001	0.695/0.305
3	14	12,918.736	12,977.713	12,933.276	0.797	0.0004	< 0.001	0.520/0.378/0.102
4	18	12,842.920	12,918.747	12,861.614	0.866	0.0017	< 0.001	0.134/0.431/0.332/0.103
5	22	12,794.636	12,887.313	12,817.484	0.855	0.4970	< 0.001	0.351/0.094/0.038/0.082/0.435

**Fig. 1:**
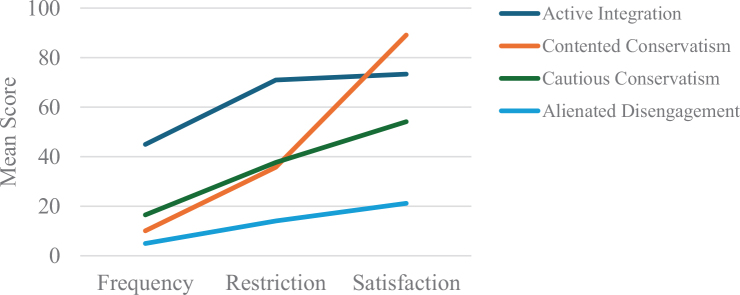
Characteristics of social participation across four profiles in stroke patients with limb dysfunction.

A graphical representation of the 4 profiles, based on mean scores of the 3e social participation dimensions, is shown in Fig. 1. The x-axis represents the social participation dimensions, and the y-axis represents the mean scores. Detailed scores and results of pairwise comparisons among profiles are provided in Table III. Each latent profile was named based on its distinctive pattern of scores across the 3 participation dimensions. Profile 1 was termed “Active Integration” (*n* = 51, 10.2%), characterized by the highest scores in participation frequency, the lowest restrictions, and relatively high satisfaction. Profile 2 was labelled “Contented Conservatism” (*n* = 67, 13.4%), exhibiting lower participation frequency, moderate restrictions, and high satisfaction levels. Profile 3, named “Cautious Conservatism” (*n* = 215, 43.1%), showed low frequency, moderate restrictions, and lower satisfaction. Profile 4 was identified as “Alienated Disengagement” (*n* = 166, 33.3%), with the lowest scores across all 3 dimensions: frequency, restriction, and satisfaction.

**Table III:** Pairwise comparison of the dimensions based on the profiles

Dimension	Scores based on the profiles (M (P25, P75))				Pairwise comparison	H	*p*-value
	C1 (Active Integration)	C2 (Contented Conservatism)	C3 (Cautious Conservatism)	C4 (Alienated Disengagement)			
Frequency	43.64 (36.36,54.55)	9.09 (3.64,14.55)	14.55 (9.09,21.82)	1.82 (0.00,9.09)	C1 > C3 > C2 > C4	244.659	< 0.001
Restriction	72.73 (48.48,90.91)	33.33 (9.09,54.55)	36.36 (24.24,51.52)	9.09 (0.00,24.24)	C1 > C2 = C3 > C4	215.679	< 0.001
Satisfaction	72.50 (70.00,77.50)	90 (82.5,95.00)	52.50 (50.00,60.00)	22.50 (15.00,30.00)	C2 > C1 > C3 > C4	420.700	< 0.001

M (P25, P75): median and interquartile range; H: value for the results of Kruskal–Wallis H test.

### Multivariate logistic analysis of factors influencing social participation profiles

Univariate analyses were conducted to examine differences in sociodemographic and clinical factors across the 4 profiles (Table IV). Variables that were statistically significant (*p* < 0.05) in univariate analyses were included as independent variables in a multinomial logistic regression model, with profile membership as the dependent variable. The results of the multivariate logistic regression are presented in Table V. Compared with the “Cautious Conservatism” group, participants with higher resilience and younger age were more likely to belong to the “Active Integration” group; older age, longer disease duration, and female sex were associated with higher likelihood of classification into the “Contented Conservatism” group; lower resilience, poorer physical function, and older age increased the probability of belonging to the “Alienated Disengagement” group.

**Table IV:** Univariate analysis of variables influencing the potential profiles of social participation

Variables	Active Integration	Contented Conservatism	Cautious Conservatism	Alienated Disengagement	X^2^/F	*p*-value
Age	55.18 ± 14.83	67.93 ± 15.62	64.67 ± 13.28	69.98 ± 8.98	19.467	< 0.001
Gender						
Male	26	32	140	100	8.224	0.042
Female	25	35	75	66		
Education						
Primary school and below	22	31	75	33	51.103	< 0.001
Middle school	16	24	112	124		
University and above	13	12	28	9		
Employed before onset						
Yes	14	16	42	23	6.288	0.098
No	37	51	173	143		
Spousal status						
Yes	43	55	185	147	1.878	0.598
No	8	12	30	19		
Care type						
No caregiver	7	9	11	2	37.741	< 0.001
Care provided by relatives	32	30	104	70		
Care provided by nursing assistant	12	28	100	94		
Family financial situation						
Poor	2	1	13	15	11.681	0.069
Average	25	47	136	94		
Good	24	19	66	57		
Stroke type						
Ischaemic	35	53	162	122	1.845	0.605
Haemorrhagic	16	14	53	44		
Onset time	9.95 ± 15.47	24.13 ± 50.95	12.23 ± 18.17	13.23 ± 17.67	4.581	0.004
Physical function						
1 point	11	10	11	3	69.776	< 0.001
2 points	24	27	80	30		
3 points	11	18	94	90		
4 points	5	12	30	43		
Resilience	27.95 ± 7.16	21.92 ± 9.45	22.17 ± 7.00	18.58 ± 8.71	19.092	< 0.001

**Table V:** Multivariate logistic regression analysis of variables influencing the potential profiles of social participation

Comparison	Variables	β	Standard error	Wald X^2^	*p*-value	OR	95% CI
χ² = 200.304, *p* < 0.001, Nagelkerke’s R² = 0.361							
Active Integration	Intercept	–2.280	1.219	3.495	0.062		
	Onset	–0.001	0.011	0.016	0.899	0.999	0.977–1.020
	Resilience	1.000	0.024	17.388	< 0.001	1.105	1.054–1.158
	Age	–0.033	0.013	6.204	0.013	0.968	0.944–0.993
	Gender = male (reference: female)	–0.368	0.366	1.012	0.315	0.692	0.338–1.418
	Care type = Unattended care (reference: By a nanny)	0.866	0.706	1.502	0.220	2.377	0.595–9.489
	Care type = By relatives (reference: By a nanny)	0.677	0.409	2.739	0.098	1.969	0.883–4.392
	Physical function = level 1 (reference: level 4)	0.662	0.739	0.804	0.370	1.939	0.456–8.248
	Physical function = level 2 (reference: level 4)	0.046	0.593	0.006	0.938	1.047	0.328–3.348
	Physical function = level 3 (reference: level 4)	–0.456	0.629	0.526	0.468	0.634	0.184–2.176
	Education condition=Primary school and below (reference: University and above)	0.741	0.527	1.981	0.159	2.099	0.748–5.890
	Education condition = Secondary school (reference: University and above)	–0.467	0.513	0.830	0.362	0.627	0.230–1.712
Contented Conservatism	Intercept	–2.543	1.102	5.329	0.021		
	Onset	0.013	0.006	4.586	0.032	1.013	1.001–1.025
	Resilience	–0.003	0.020	0.020	0.886	0.997	0.959–1.037
	Age	0.030	0.013	5.301	0.021	1.031	1.004–1.057
	Gender = male (reference: female)	–0.627	0.306	4.195	0.041	0.534	0.293–0.973
	Care type = Unattended care (reference: By a nanny)	0.855	0.604	2.000	0.157	2.351	0.719–7.689
	Care type = By relatives (reference: By a nanny)	0.187	0.322	0.337	0.562	1.206	0.641–2.266
	Physical function = level 1 (reference: level 4)	0.966	0.631	2.340	0.126	2.627	0.762–9.055
	Physical function = level 2 (reference: level 4)	0.052	0.452	0.013	0.908	1.053	0.434–2.557
	Physical function = level 3 (reference: level 4)	–0.408	0.456	0.797	0.372	0.665	0.272–1.628
	Education condition = Primary school and below (reference: University and above)	–0.400	0.499	0.642	0.423	0.670	0.252–1.784
	Education condition = Secondary school (reference: University and above)	–0.832	0.479	3.015	0.083	0.435	0.170–1.113
Alienated Disengagement	Intercept	–0.608	0.934	0.424	0.515		
	Onset	0.003	0.006	0.210	0.647	1.003	0.991–1.014
	Resilience	–0.052	0.015	11.651	0.001	0.949	0.921–0.978
	Age	0.029	0.011	6.372	0.012	1.029	1.006–1.052
	Gender = male (reference: female)	–0.411	0.239	2.949	0.086	0.663	0.415–1.060
	Care type = Unattended care (reference: By a nanny)	–0.661	0.832	0.631	0.427	0.517	0.101–2.637
	Care type = By relatives (reference: By a nanny)	–0.169	0.235	0.513	0.474	0.845	0.532–1.340
	Physical function = level 1 (reference: level 4)	–0.981	0.750	1.712	0.191	0.375	0.086–1.630
	Physical function = level 2 (reference: level 4)	–0.989	0.355	7.749	0.005	0.372	0.186–0.746
	Physical function = level 3 (reference: level 4)	–0.273	0.303	0.812	0.368	0.761	0.420–1.379
	Education condition = Primary school and below (reference: University and above)	–0.593	0.497	1.421	0.233	0.553	0.209–1.465
	Education condition = Secondary school (reference: University and above)	0.617	0.450	1.878	0.171	1.853	0.767–4.480

The Cautious Conservatism profile was designated as the reference group. OR: odds ratio; CI: confidence interval.

### Qualitative findings

A total of 16 participants were interviewed, reaching data saturation. The sample consisted of 12 males and 4 females, with a mean age of 64.38 years (SD = 12.40). General characteristics of the participants are presented in Table VI. Qualitative analysis identified 5 overarching themes related to behavioural patterns and experiences of social participation among stroke patients.

**Table VI:** General characteristics of the participants included in the qualitative study (*n* = 16)

No.	Gender	Age	Education	Marriage	Employment before stroke	Caregiver	Family financial situation	Stroke type	Disease course	mRS
P1	Male	80	Technical Secondary School	Married	Retired	Relatives and nursing assistant	Good	Ischaemic	2.5 years	3
P2	Male	67	High School	Married	Retired	Relatives	Average	Ischaemic	10 years	1
P3	Male	65	Middle School	Married	Retired	Relatives	Average	Ischaemic	3 years	2
P4	Female	72	Middle School	Widowed	Retired	No one	Average	Ischaemic	2 years	2
P5	Male	75	Primary School	Married	Retired	Relatives	Good	Ischaemic	2 years	1
P6	Male	80	Middle School	Married	Retired	Relatives	Good	Ischaemic	1 year	1
P7	Male	70	Middle School	Married	Retired	No one	Average	Ischaemic	7 months	1
P8	Male	72	High School	Married	Retired	Relatives	Good	Ischaemic	11 years	1
P9	Male	34	University	Married	Employed	Relatives	Average	Haemorrhagic	3 years	1
P10	Male	71	High School	Married	Retired	No one	Good	Ischaemic	2 years	1
P11	Female	73	Technical Secondary School	Married	Retired	Relatives	Good	Ischaemic	3.5 years	2
P12	Male	66	Middle School	Married	Retired	No one	Average	Ischeamic	10 years	2
P13	Male	56	Middle School	Married	Employed	Relatives	Good	Haemorrhagic	6 years	2
P14	Female	79	Middle School	Widowed	Retired	No one	Average	Ischaemic	3 years	1
P15	Male	65	University	Married	Retired	Relatives	Good	Ischaemic	1 years	3
P16	Female	45	High School	Married	Employed	Relatives	Good	Haemorrhagic	3 months	4

### Behavioural patterns in patients’ social participation

Three primary behavioural patterns emerged from the data regarding how patients approached social participation.

The activist faction referred to the behavioural strategy of stroke patients with limb dysfunction who, under the influence of various factors, actively sought social participation and faced life’s challenges with a positive attitude. They usually took the initiative to engage in social activities, strived to overcome physical barriers, and worked to integrate into society. As 1 participant described it:

Not long after I got sick and came home, I went downstairs by myself. I live on the 4th floor. [Researcher: Was someone accompanying you?] No, who would accompany me? People were afraid to accompany me, worried that if something happened to me, I would sue them for compensation. I relied on myself. Once I could walk around the house by myself, I tried going downstairs. Sometimes, when people kindly saw me and offered to carry things for me, I usually refused. I just relied on myself. If I could move by myself, I would do it on my own.

These participants generally possessed higher levels of resilience, enabling them to adapt more quickly to the challenges brought about by illness. They were not overwhelmed by setbacks and difficulties but were able to adjust their mindset swiftly and face life’s challenges with a positive attitude. Additionally, they tended to be stronger in personality traits and more optimistic in their outlook. They were inclined to rely on their own strength to solve problems. Moreover, this group often benefited from more robust family and social support systems, gaining favourable economic and emotional support as well as abundant social resources and opportunities.

Conservatives were stroke patients with limb dysfunction who, under the influence of various factors, tended to adopt a more cautious and risk-averse behavioural strategy. They usually tried to minimize going out and social activities to avoid potential physical discomfort or accidental risks. These patients often included the ability to participate in social activities, but expressed significant psychological concerns. As P4 explained:

I don’t really dare to go out. Previously, we used to walk by the river every day, greeting each other. Later, she didn’t show up for three days, and they said she had passed away and was already cremated. So, I don’t really dare to go out now and just stay active in the neighbourhood.

This patient, having experienced the sudden death of a companion, became more concerned about her health and preferred to limit her social activities to the immediate community. The spouse of P8 added:

*He once walked too fast and fell, knocking out a tooth. He was terrified. So now he walks very slowly, one step at a time, as if he’s stepping on ants [P8: It’s okay to walk slowly], very slowly, like the way elderly people walk.*

Another spouse noted: “He has fallen three times before, but fortunately, he didn’t fracture any bones. So now we are very careful and don’t let him go out alone” (P1’s spouse).

Stroke patients with limb dysfunction frequently experienced poor balance, weak muscle strength, and instability, which increased the risk of falling. Many stroke patients, especially those with prior fall experiences, became more conservative in their social activities, prioritizing safety while maintaining their physical functions. Patients with this behavioural strategy was influenced not only by their physical func-tions but also by other factors, such as negative past experiences in social participation, lack of confidence in their ability to engage socially, overprotection from family members, and unfavourable environmental conditions.

The alienated group comprised stroke patients with physical disabilities who, due to a multitude of factors, experienced a decrease in motivation and willingness to participate socially, maintaining a low-intensity, static level of engagement. “Since getting sick, I haven’t used my mobile phone or computer, cutting off my connection with the outside world” (P2). The alienated group was closely related to the impaired physical functions. The physical limitations such as inconvenience in movement and restricted activity capabilities made patients experience a sense of loss of control and feel incapable when facing social participation, thus reducing their motivation and willingness to engage. Additionally, changes in body image may impacted their self-esteem, thereby reducing their willingness to engage in social activities and leading them to actively sever social interactions. This pattern also correlated with lower resilience levels; these patients often lacked sufficient adaptive capacity to actively cope with life’s changes and challenges, thus causing them to prefer an avoidant way of living. Furthermore, it may also be related to inadequate family and social support, as well as unfavourable environmental conditions.

#### Emotional experience of patients’ social participation

Sense of loss described the complex emotional experience of stroke patients with physical disabilities, who, when reviewing and comparing their social participation before and after the onset of the disease, subjectively perceived a significant decrease in their ability or opportunity to fulfil roles, engage in activities, connect with others, and achieve self-worth across multiple dimensions. This led to a profound and persistent feeling of longing and loss. The essence of the sense of loss was that these patients habitually compared their current state with the period before the stroke. Therefore, when asked about their views on current social participation, these patients often expressed that “It’s incomparable to before the illness; I can’t do XX anymore” (for example, P3 described I thus: “This condition greatly affects life, there’s no way around it, now I can’t even travel”). While decreased social participation following stroke represented an objective reality for most patients with physical disabilities, continuously making such comparisons intensified their sorrowful feelings and hindered current social engagement. As P8’s spouse recounted, “He has nothing much to do, just sits on the sofa, and when I ask if he wants to go out, he gets very irritable and says, ‘I’m not in the mood today, I don’t want to go out’”.

The sense of loss appeared closely related to the rigid self-identity of stroke patients with physical disabilities and the absence of downward comparison. In psychology, self-identity refers to an individual’s understanding and perception of who they are, and what traits, values, and goals they possess (27). It is an important part of self-awareness and reflects a person’s internal perception of their identity (28). When stroke patients with physical disabilities tightly bound their self-concept to the “intact” state before the illness, it became relatively more difficult to integrate the “disabled” state after the illness into a new, equally valuable self-identity. These patients demonstrated limited identification and trust in their “disabled” self, and thus showed a lack of interest in social participation across multiple dimensions such as role fulfilment, activity range, interpersonal connections, and self-worth achievement. As P1’s spouse said, “He seems to have lost interest in these activities”. The second possible reason for the sense of loss involved the absence of downward comparison. Downward social comparison is an important concept in social comparison theory, first proposed by psychologist Leon Festinger in 1954 (29). It refers to individuals comparing themselves with those who are doing worse, thereby enhancing their self-esteem and self-evaluation. When stroke patients with physical disabilities engaged in effective downward comparison, such as recognizing their own survival and considering others in worse conditions, it helped alleviate negative emotions and strengthen confidence in dealing with setbacks. As P11 described it, “I went to the rehabilitation hospital for recovery, and there were many people in worse conditions than me.” However, when patients lacked downward comparison, they were more likely to remain stuck in a rigid self-identity, leading to a more severe sense of loss in social participation.

The sense of rebuilding captured the positive psychological experience of stroke patients with physical disabilities, who, after experiencing the significant impact of the disease, through continuous efforts, adaptation, and adjustment, compared their current social participation with the state immediately after the onset of the disease. They subjectively perceived a significant improvement in their ability or opportunity to fulfil roles, engage in activities, connect with others, and achieve self-worth across multiple dimensions. The essence of the sense of rebuilding involved comparing the current social participation with the period of most severe functional impairment (usually immediately after the onset or the acute phase). Therefore, when asked about their views on current social participation, patients often expressed that “It’s much better than when I first got sick”; such as when P13 said:

*At first, the doctor told me that I might have to lie in bed for the rest of my life, but now I have recovered well enough to walk, although I’m not stable and have poor balance, but I have recovered quite well. Last year, I was even cooking, but this year, because the stove was slippery, I can’t cook with one hand.*

Although this sense of rebuilding does not necessarily require the patient’s physical function to recover to a normal level, and the patient may still have irreversible physical function damage and lifelong disability, comparing with the worst state after the disease onset helped the patient face current social life with a positive attitude. “When I first got out of the hospital, I couldn’t do anything, but now I can walk with a limp and even cook for myself, one meat dish and one vegetable dish, taking care of my spouse, all by myself” (P7).

The possible reason for the sense of rebuilding was the patient’s acceptance of the disease. After experiencing a significant event, patients who accepted reality acknowledged disability as their new reality and discovered new meaning and possibilities based on this acceptance. These patients were more inclined to focus on the present, shifting their goals from “returning to the past” to “achieving the best functional state”. They were more likely to take proactive approaches to explore social activities they can participate in with their current abilities.

*Researcher: You just described facing many difficulties when going downstairs. Can you describe how you feel about it? “It’s all about mindset, it depends on yourself, that’s enough. Without a strong will, what do you have? What’s the point of living? You have to have flavour, life has to have flavour, that’s it.” (P7)*

Despite having physical disabilities (mRS score of 2) and needing to care for a spouse with a mental disorder who is bedridden, this research subject did not complain about his current situation during the 72-min interview. His use of the word “flavour,” full of warmth, to describe his life, demonstrated both his acceptance of the stroke and his efforts to return to society in his own way.

The sense of loss and the sense of rebuilding are best understood not as a binary dichotomy, but as anchoring a spectrum of emotional experiences of social participation. They could coexist in the same individual in varying degrees and change with different stages of the disease process. The sense of loss constituted a natural reaction to the catastrophic event of stroke, but when it persisted and solidified, it became a major obstacle to social participation. In contrast, the sense of rebuilding reflected the patient’s resilience and adaptability, representing a positive subjective experience of social participation.

#### Quantitative–qualitative integration

**Fig. 2:**
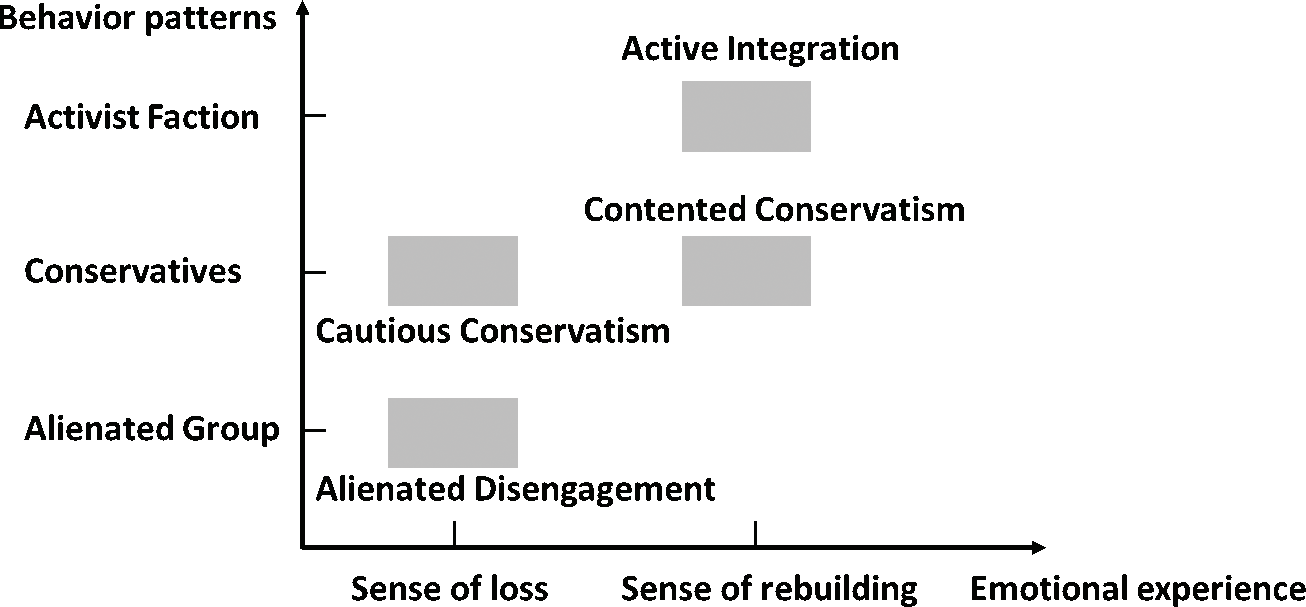
Quantitative–qualitative integration.

The integration of quantitative and qualitative findings is summarized in Fig. 2. Quantitatively, the “Active Integration” profile was characterized by high participation frequency, low restrictions, and high satisfaction, which aligned cohesively with the qualitative activist faction behavioural pattern and was predominantly guided by a sense of rebuilding. The qualitative analysis defined the sense of rebuilding as an emotional experience characterized by acceptance of reality, and a positive mindset – an internal state logically associated with higher social participation satisfaction. A particularly revealing contrast emerged between the “Contented Conservatism” and “Cautious Conservatism” profiles. While both exhibited similar moderate participation frequency and restrictions and corresponded to the conservatives behavioural pattern, they diverged fundamentally in their emotional drivers and resulting satisfaction. The high satisfaction in the “Contented Conservatism” profile was interpreted as being underpinned by a sense of rebuilding, explaining how individuals achieved contentment within a limited scope of activities. In contrast, the lower satisfaction in the “Cautious Conservatism” profile was linked to a sense of loss (characterized by grief over functional decline and a focus on the pre-stroke self), which is logically associated with diminished satisfaction. The “Alienated Disengagement” profile, defined by very low frequency, very high restrictions, and very low satisfaction, formed a coherent cluster with the qualitative alienated group behavioural pattern and a dominant sense of loss. In fact, the emotional experiences of stroke patients are distributed along a continuum between a sense of rebuilding and a sense of loss. Although Fig. 2 only marks these 2 endpoints on the horizontal axis, we conceptualize patients’ emotional positioning across this spectrum. Specifically, individuals with higher satisfaction scores (Active Integration and Contented Conservatism) are positioned closer to the sense of rebuilding, while those with lower satisfaction (Cautious Conservatism and Alienated Disengagement) are located nearer to the sense of loss.

## DISCUSSION

This study identified 4 distinctive profiles of social participation of stroke survivors with limb dysfunction through LPA (Active Integration, Contented Conservatism, Cautious Conservatism, and Alienated Disengagement), and through qualitative research we deeply depicted the unique behavioural patterns (activist faction, conservatives, and alienated group) and dominant emotional experiences (sense of rebuilding and sense of loss) of each profile. This study was one of the first to assess in depth the heterogeneity and internal experiences of social participation of this group within the Chinese cultural context using a mixed-method approach. The integration of quantitative and qualitative results provides a more comprehensive and multi-faceted perspective for understanding the complexity of social participation.

Our findings indicate that 10.2% of survivors, categorized as “Active Integration”, maintain a relatively high frequency of social participation and report positive participatory experiences. Qualitative data reveal that these individuals tend to engage proactively in social activities and perceive their current involvement more favourably by comparing it with their condition shortly after diagnosis. Notably, another 13.4% of participants, classified as “Contented Conservatism”, report high satisfaction with social participation despite experiencing noticeable restrictions. Qualitative insights suggest that this contentment stems largely from a feeling of “rebuilding”, consistent with previous studies indicating that the quality (rather than the frequency or quantity) of social participation is central to subjective experience (30). These results carry important clinical implications: for this subgroup of stroke survivors, interventions should emphasize enhancing the meaning and quality of social engagement rather than merely increasing the number of activities. Rather than striving to expand the range of participative acts, support efforts should focus on helping individuals accept the irreversible aspects of their disability and identify forms of participation that align with their current needs and values (31). This person-centred, quality-oriented approach represents a shift beyond conventional intervention strategies that have traditionally prioritized quantitative increases in social activity.

Furthermore, this study revealed that 43.1% of stroke survivors (Cautious Conservatism) exhibited moderate levels of social participation restriction, frequency, and satisfaction. In contrast, 33.3% of survivors (Alienated Disengagement) showed high levels of restriction alongside low frequency and satisfaction. These results indicate that the majority of stroke survivors with physical disabilities experience relatively limited social participation and report unsatisfactory participatory experiences (32). Importantly, impaired social participation cannot be attributed solely to physical dysfunction; it is also closely associated with participants’ engagement strategies and psychological attitudes. Consequently, clinical interventions should not only focus on physical function training, such as virtual reality (33), robotic technology (34), and other rehabilitation devices (35, 36), but must also prioritize psychological adjustment. Enhancing motivation, fostering a positive attitude toward social reintegration, and supporting survivors in actively engaging with post-disability social life are critical components of effective rehabilitation (37, 38). It is particularly noteworthy that while quantitative measures of participation behaviour were similar between the “Contented Conservatism” and “Cautious Conservatism” groups, their levels of satisfaction differed significantly. Qualitative results identified that a sense of rebuilding vs a sense of loss served as the key differentiator influencing satisfaction. The sense of rebuilding reflects the patients’ acceptance of their disease and disability, which can serve as a powerful psychological resource (39, 40), enabling individuals to adopt a constructive attitude towards social life, for example by actively seeking help, thereby expanding opportunities and conditions for participation. The 4-profile classification also suggests that improving social participation does not necessarily require all patients to achieve the highest engagement level. Instead, a progressive, profile-based approach is recommended, for instance, supporting transition from Alienated Disengagement to Cautious Conservatism, or from Contented Conservatism to Active Integration. These findings provide a valuable evidence base for developing targeted clinical interventions tailored to patients’ specific social participation patterns.

This study revealed that individuals with higher levels of resilience were more likely to belong to the “Active Integration” profile, whereas those with lower resilience tended to be classified as “Alienated Disengagement”. However, resilience levels did not significantly influence classification into the “Contented Conservatism” or “Cautious Conservatism” profiles. These findings align with previous research supporting a positive correlation between resilience and social participation (41). Resilience is conceptualized as a dynamic process of positive adaptation in the face of adversity or trauma (42). Previous studies indicate that individuals with greater resilience are better equipped to mobilize internal and external resources, facilitating rapid recovery from negative experiences and buffering the impact of stressors (43). Recent evidence further suggests that resilience facilitates neuroplasticity and adaptive processes through the distinct yet synergistic actions of catecholamines (such as dopamine and norepinephrine) and glucocorticoids (44). In the context of stroke recovery, survivors with higher resilience demonstrate an enhanced capacity to adapt to disability-related changes and challenges. These individuals demonstrate a proactive approach and achieve higher levels of social engagement. Crucially, they also derive genuine satisfaction from their social experiences. Therefore, resilience should be considered a key target for interventions aimed at improving social participation in stroke patients with physical disabilities.

The study also found that, compared with the Cautious Conservatism profile, individuals with poorer limb function were more likely to be classified into the Alienated Disengagement profile, while limb function did not significantly influence classification into the remaining categories. This finding both aligns with and interestingly extends a substantial body of prior research emphasizing that “physical function is a key determinant of social participation” (45, 46). First, significant deterioration in limb function may act as the “final straw” that overwhelms a patient’s willingness to participate socially, pushing them into a state of Alienated Disengagement. When functional impairment reaches a certain severity (e.g., mRS grade 4), it creates an almost insurmountable objective barrier, rendering most social activities physically “impossible” or “extremely difficult”, thereby directly causing a sharp decline in both participation frequency and satisfaction. This aligns with the classical paradigm established in previous studies. However, more importantly, limb function did not significantly distinguish among the Active Integration, Contented Conservatism, and Cautious Conservatism profiles. This suggests that physical capacity is not the dominant differentiating factor in social participation patterns until a certain threshold of severe disability is reached. Instead, psychological and emotional factors likely play a more central role than objective physical function. Therefore, the findings of this study refine the traditional linear understanding that better function leads to better participation and propose a more nuanced model: Limb function is a prerequisite for social participation, but it alone is not sufficient to determine its nature or quality. It plays a decisive role when impairment is severe (defining the lower bound of participation), but within the range of moderate dysfunction, individual psychological and cognitive factors (47), such as resilience, comparison strategies, and self-identity, serve as the critical watershed that determines whether one ultimately follows an Active, Contented, or Cautious path.

Furthermore, this study identified age as a significant factor differentiating social participation profiles. Younger age predicted membership in the Active Integration profile, whereas older age increased the likelihood of Contented Conservatism or Alienated Disengagement, consistent with existing literature (48). Physiologically, the greater resilience and neuroplasticity of younger patients support faster recovery, enabling active reintegration. In contrast, older adults often face comorbidities and functional decline, predisposing them to disengagement (49). Social roles also contribute: younger individuals are often driven by familial or occupational duties, while older adults, frequently retired, may either adapt contentedly to reduced roles or experience significant loss (50). Rehabilitation strategies should thus be age-specific: supporting younger patients in reclaiming social roles, and assisting older adults in constructing new, meaningful forms of participation. Additionally, this study found that females were more likely to belong to the Contented Conservatism profile than to Cautious Conservatism, which contrasts with previous reports of no significant gender differences in participation satisfaction (51). This discrepancy may reflect traditional gender roles in Chinese society, where women are often socialized to prioritize family harmony. Such expectations may encourage emotion regulation, cognitive reframing, and downward comparison, enabling them to find satisfaction in manageable activities like household tasks.

### Limitations

Several limitations should be acknowledged. First, the cross-sectional design precludes establishing causal relationships between the identified predictors and profile membership. Longitudinal studies are needed to examine the stability of these profiles and their trajectories over time. Second, the generalizability of the findings may be limited as participants were recruited solely from rehabilitation centres in Shanghai, and the qualitative sample exhibited a gender imbalance (with more males), which may not fully represent potential gender-specific experiences. Future multi-centre studies with more diverse sampling are warranted. Third, the exclusion of patients with cognitive impairments and communicative disorders may limit the generalizability of our findings to the broader stroke population. Fourth, although a mixed-methods approach was employed, the potential for common method bias in the self-reported quantitative data remains. Finally, environmental factors, such as community accessibility and social policies, were not quantitatively assessed and integrated into the predictive model, representing an important avenue for future research. Specifically, future studies could quantitatively investigate the impact of specific policies (e.g., long-term care insurance) and built environment factors on social participation profiles, employing longitudinal or multi-centre designs.

### Conclusion

This study employed a convergent mixed-methods design to systematically reveal, for the first time, 4 heterogeneous latent profiles of social participation among Chinese stroke patients with limb dysfunction: “Active Integration”, “Contented Conservatism”, “Cautious Conservatism”, and “Alienated Disengagement”. Each profile is distinguished not only by objective manifestations of social participation (frequency, restrictions) but also by unique primary behavioural patterns (active, conservative, alienated) and dominant emotional experiences (sense of rebuilding, sense of loss). Clinical practitioners should acknowledge this heterogeneity and adopt a precision strategy of “tailored intervention based on profiling” and “step-by-step enhancement” to effectively improve patients’ overall level of social participation and quality of life.
